# 10-Hy­droxy­benzo[*h*]quinolin-1-ium tetra­chlorido(pyridine-2-carboxyl­ato-κ^2^ *N*,*O*)stannate(IV) methanol monosolvate

**DOI:** 10.1107/S1600536812019605

**Published:** 2012-05-05

**Authors:** Ezzatollah Najafi, Mostafa M. Amini, Seik Weng Ng

**Affiliations:** a Department of Chemistry, General Campus, Shahid Beheshti University, Tehran 1983963113, Iran; b Department of Chemistry, University of Malaya, 50603 Kuala Lumpur, Malaysia; c Chemistry Department, Faculty of Science, King Abdulaziz University, PO Box 80203 Jeddah, Saudi Arabia

## Abstract

The reaction of 4-(dimethyl­amino)­pyridine, picolinic acid and stannic chloride yields the title monosolvated salt, (C_13_H_10_NO)[SnCl_4_(C_6_H_4_NO_2_)]·CH_3_OH. The Sn^IV^ atom is *N*,*O*-chelated by the picolinate ion in a *cis*-SnNOCl_4_ octa­hedral geometry. The cation is linked to the methanol solvent mol­ecule by an O—H⋯O hydrogen bond; the solvent mol­ecule itself is a hydrogen-bond donor to the uncoordinating carboxyl­ate O atom of the anion. The cations and anions are linked by weak N—H⋯Cl inter­actions, forming a chain running along the *b* axis.

## Related literature

For a tetra­chlorido(pyridine-2-carboxyl­ato)stannate(IV), see: Najafi *et al.* (2012 ▶).
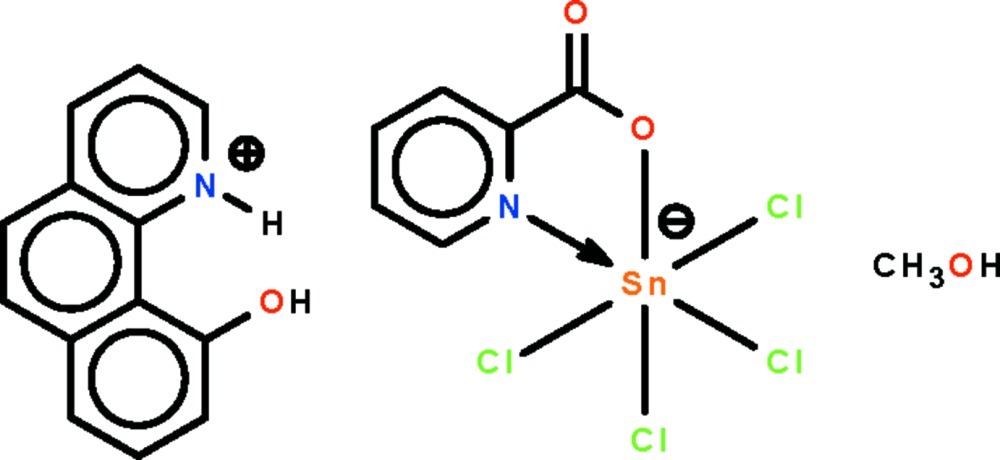


## Experimental

### 

#### Crystal data

(C_13_H_10_NO)[SnCl_4_(C_6_H_4_NO_2_)]·CH_4_O
*M* *_r_* = 610.85
Monoclinic, 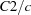

*a* = 31.5065 (11) Å
*b* = 8.0802 (2) Å
*c* = 20.0948 (9) Å
β = 115.722 (5)°
*V* = 4608.8 (3) Å^3^
*Z* = 8
Mo *K*α radiation
μ = 1.60 mm^−1^
*T* = 100 K
0.30 × 0.20 × 0.10 mm

#### Data collection

Agilent SuperNova Dual diffractometer with an Atlas detector
Absorption correction: multi-scan (*CrysAlis PRO*; Agilent, 2012) ▶ *T*_min_ = 0.645, *T*_max_ = 0.856
15316 measured reflections
5326 independent reflections
4445 reflections with *I* > 2σ(*I*)
*R*_int_ = 0.033

#### Refinement

*R*[*F* ^2^ > 2σ(*F* ^2^)] = 0.028
*wR*(*F* ^2^) = 0.065
*S* = 1.02
5326 reflections
293 parameters
3 restraints
H atoms treated by a mixture of independent and constrained refinement
Δρ_max_ = 0.51 e Å^−3^
Δρ_min_ = −0.44 e Å^−3^

### 

Data collection: *CrysAlis PRO* (Agilent, 2012 ▶); cell refinement: *CrysAlis PRO*; data reduction: *CrysAlis PRO*; program(s) used to solve structure: *SHELXS97* (Sheldrick, 2008 ▶); program(s) used to refine structure: *SHELXL97* (Sheldrick, 2008 ▶); molecular graphics: *X-SEED* (Barbour, 2001 ▶); software used to prepare material for publication: *publCIF* (Westrip, 2010 ▶).

## Supplementary Material

Crystal structure: contains datablock(s) global, I. DOI: 10.1107/S1600536812019605/hg5222sup1.cif

Structure factors: contains datablock(s) I. DOI: 10.1107/S1600536812019605/hg5222Isup2.hkl

Additional supplementary materials:  crystallographic information; 3D view; checkCIF report

## Figures and Tables

**Table 1:** Hydrogen-bond geometry (Å, °)

*D*—H⋯*A*	*D*—H	H⋯*A*	*D*⋯*A*	*D*—H⋯*A*
O3—H3⋯O4	0.84 (1)	1.73 (1)	2.554 (2)	170 (3)
O4—H4⋯O2	0.83 (1)	1.87 (1)	2.698 (3)	174 (3)
N2—H2⋯Cl4^i^	0.87 (1)	2.58 (2)	3.255 (2)	135 (2)

Symmetry code: (i) 
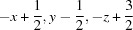
.

**Fig. 1.**
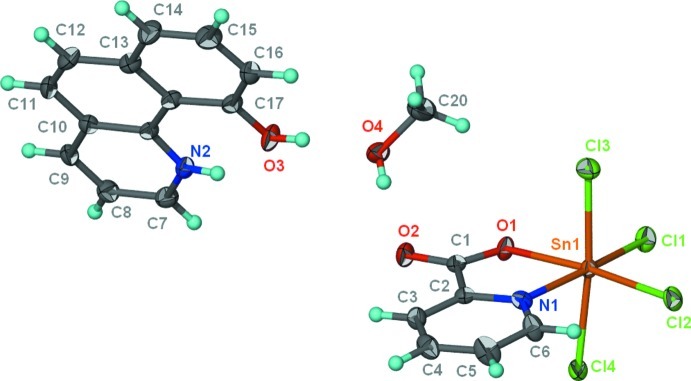
Thermal ellipsoid plot (Barbour, 2001) of (C13H10NO)[SnCl4(C6H4NO2)].CH3OH at the 70% probability level; hydrogen atoms are drawn as spheres of arbitrary radius.

## supplementary crystallographic information

### Comment

A previous study reported 4-(dimethylamino)pyridinium tetrachlorido(pyridine-2-carboxylato)stannate, which was synthesized by the reaction of 4-(dimethylamino)pyridine, picolinic acid and stannic chloride in methanol (Najafi *et al.*, 2012). The reaction with 10-hydroxybenzo[*h*]quinoline in place of 4-(dimethylamino)pyridine yielded the analogous salt, (C_13_H_10_NO)[SnCl_4_(C_6_H_4_NO_2_)] as a methanol monosolvate (Scheme I). The Sn^IV^ atom is *N*,*O*-chelated by the picolinate ion in a *cis*-SnNOCl_4_ octahedral geometry. The cation is linked to the methanol molecule by an O–H···O hydrogen bond; the solvent molecule itself is hydrogen-bond donor to the double-bond carboxyl O atom of the anion (Table 1).

### Experimental

Stannic chloride pentahydrate (0.35 g, 1 mmol), picolinic acid (0.12 g, 1 mmol) and 10-hydroxybenzo[*h*]quinoline (0.20 g, 1 mmol) were loaded into a convection tube; the tube was filled with dry methanol and kept at 333 K. Yellow crystals were collected from the side arm after several days.

### Refinement

Carbon-bound H-atoms were placed in calculated positions [C–H 0.95 to 0.98 Å, *U*_iso_(H) 1.2 to 1.5*U*_eq_(C)] and were included in the refinement in the riding model approximation.

The hydroxy and ammonium H-atoms were located in a difference Fourier map, and were refined with distance restraints of O–H 0.84±0.01 and N–H 0.88±0.01 Å; their temperature factors were was refined.

### Crystal data

**Table:**

(C_13_H_10_NO)[SnCl_4_(C_6_H_4_NO_2_)]·CH_4_O	*F*(000) = 2416
*M**_r_* = 610.85	*D*_x_ = 1.761 Mg m^−^^3^
Monoclinic, *C*2/*c*	Mo *K*α radiation, λ = 0.71073 Å
Hall symbol: -C 2yc	Cell parameters from 6804 reflections
*a* = 31.5065 (11) Å	θ = 2.6–27.5°
*b* = 8.0802 (2) Å	µ = 1.60 mm^−^^1^
*c* = 20.0948 (9) Å	*T* = 100 K
β = 115.722 (5)°	Prism, yellow
*V* = 4608.8 (3) Å^3^	0.30 × 0.20 × 0.10 mm
*Z* = 8	

### Data collection

**Table:**

Agilent SuperNova Dual diffractometer with an Atlas detector	5326 independent reflections
Radiation source: SuperNova (Mo) X-ray Source	4445 reflections with *I* > 2σ(*I*)
Mirror monochromator	*R*_int_ = 0.033
Detector resolution: 10.4041 pixels mm^-1^	θ_max_ = 27.6°, θ_min_ = 2.6°
ω scan	*h* = −29→40
Absorption correction: multi-scan (*CrysAlis PRO*; Agilent, 2012)	*k* = −10→10
*T*_min_ = 0.645, *T*_max_ = 0.856	*l* = −26→16
15316 measured reflections	

### Refinement

**Table:**

Refinement on *F*^2^	Primary atom site location: structure-invariant direct methods
Least-squares matrix: full	Secondary atom site location: difference Fourier map
*R*[*F*^2^ > 2σ(*F*^2^)] = 0.028	Hydrogen site location: inferred from neighbouring sites
*wR*(*F*^2^) = 0.065	H atoms treated by a mixture of independent and constrained refinement
*S* = 1.02	*w* = 1/[σ^2^(*F*_o_^2^) + (0.0262*P*)^2^ + 2.9883*P*] where *P* = (*F*_o_^2^ + 2*F*_c_^2^)/3
5326 reflections	(Δ/σ)_max_ = 0.001
293 parameters	Δρ_max_ = 0.51 e Å^−^^3^
3 restraints	Δρ_min_ = −0.44 e Å^−^^3^

### Fractional atomic coordinates and isotropic or equivalent isotropic displacement parameters (Å^2^)

**Table:**

	*x*	*y*	*z*	*U*_iso_*/*U*_eq_	
Sn1	0.341644 (5)	0.660077 (19)	0.666671 (10)	0.01289 (6)	
Cl1	0.37922 (2)	0.39689 (7)	0.68079 (4)	0.02026 (14)	
Cl2	0.40850 (2)	0.82509 (7)	0.68449 (4)	0.02084 (15)	
Cl3	0.31336 (2)	0.66098 (8)	0.53578 (4)	0.02510 (15)	
Cl4	0.36197 (2)	0.66555 (8)	0.79760 (4)	0.02179 (15)	
O1	0.27693 (5)	0.5575 (2)	0.65110 (10)	0.0169 (4)	
O2	0.20485 (6)	0.6113 (2)	0.64027 (11)	0.0225 (4)	
O3	0.07905 (6)	0.2706 (2)	0.53486 (11)	0.0217 (4)	
O4	0.16051 (6)	0.3577 (2)	0.54923 (12)	0.0238 (4)	
N1	0.29836 (7)	0.8782 (2)	0.66282 (12)	0.0144 (4)	
N2	0.02661 (7)	0.1368 (3)	0.58997 (13)	0.0172 (5)	
C1	0.24389 (8)	0.6557 (3)	0.64886 (14)	0.0153 (5)	
C2	0.25594 (8)	0.8378 (3)	0.65863 (14)	0.0148 (5)	
C3	0.22558 (9)	0.9549 (3)	0.66296 (15)	0.0191 (6)	
H3A	0.1957	0.9238	0.6599	0.023*	
C4	0.23938 (9)	1.1189 (3)	0.67188 (16)	0.0215 (6)	
H4A	0.2192	1.2022	0.6753	0.026*	
C5	0.28285 (10)	1.1601 (3)	0.67575 (18)	0.0253 (7)	
H5	0.2928	1.2723	0.6813	0.030*	
C6	0.31165 (9)	1.0370 (3)	0.67148 (16)	0.0209 (6)	
H6	0.3418	1.0654	0.6748	0.025*	
C7	0.02266 (9)	0.0482 (3)	0.64277 (15)	0.0200 (6)	
H7	0.0501	0.0137	0.6846	0.024*	
C8	−0.02144 (9)	0.0061 (3)	0.63690 (16)	0.0207 (6)	
H8	−0.0246	−0.0580	0.6741	0.025*	
C9	−0.06052 (9)	0.0593 (3)	0.57607 (16)	0.0192 (6)	
H9	−0.0910	0.0315	0.5714	0.023*	
C10	−0.05622 (9)	0.1538 (3)	0.52073 (15)	0.0173 (5)	
C11	−0.09634 (9)	0.2137 (3)	0.45798 (16)	0.0214 (6)	
H11	−0.1271	0.1894	0.4528	0.026*	
C12	−0.09079 (9)	0.3046 (3)	0.40608 (16)	0.0228 (6)	
H12	−0.1180	0.3440	0.3650	0.027*	
C13	−0.04506 (9)	0.3436 (3)	0.41098 (15)	0.0191 (6)	
C14	−0.04056 (9)	0.4373 (3)	0.35614 (16)	0.0236 (6)	
H14	−0.0680	0.4748	0.3149	0.028*	
C15	0.00334 (9)	0.4759 (3)	0.36123 (16)	0.0240 (6)	
H15	0.0059	0.5400	0.3235	0.029*	
C16	0.04405 (9)	0.4220 (3)	0.42113 (16)	0.0208 (6)	
H16	0.0741	0.4495	0.4240	0.025*	
C17	0.04076 (9)	0.3285 (3)	0.47630 (15)	0.0172 (5)	
C18	−0.00405 (9)	0.2878 (3)	0.47287 (15)	0.0166 (5)	
C19	−0.01061 (8)	0.1925 (3)	0.52815 (15)	0.0153 (5)	
C20	0.18022 (11)	0.3508 (4)	0.49717 (18)	0.0330 (7)	
H20A	0.1712	0.4503	0.4662	0.050*	
H20B	0.1683	0.2525	0.4659	0.050*	
H20C	0.2146	0.3448	0.5237	0.050*	
H2	0.0542 (6)	0.171 (3)	0.5956 (17)	0.027 (8)*	
H3	0.1041 (7)	0.306 (4)	0.5351 (19)	0.042 (10)*	
H4	0.1730 (9)	0.434 (2)	0.5790 (13)	0.021 (8)*	

### Atomic displacement parameters (Å^2^)

**Table:**

	*U*^11^	*U*^22^	*U*^33^	*U*^12^	*U*^13^	*U*^23^
Sn1	0.01130 (9)	0.01287 (9)	0.01497 (11)	−0.00094 (7)	0.00613 (7)	−0.00093 (7)
Cl1	0.0175 (3)	0.0155 (3)	0.0278 (4)	0.0006 (3)	0.0098 (3)	−0.0006 (3)
Cl2	0.0182 (3)	0.0206 (3)	0.0289 (4)	−0.0060 (3)	0.0151 (3)	−0.0059 (3)
Cl3	0.0224 (3)	0.0360 (4)	0.0157 (4)	0.0055 (3)	0.0071 (3)	−0.0004 (3)
Cl4	0.0147 (3)	0.0370 (4)	0.0143 (3)	0.0024 (3)	0.0068 (3)	0.0010 (3)
O1	0.0114 (8)	0.0143 (8)	0.0242 (11)	−0.0025 (7)	0.0072 (8)	−0.0018 (7)
O2	0.0135 (9)	0.0271 (10)	0.0276 (12)	−0.0053 (8)	0.0096 (9)	−0.0072 (9)
O3	0.0120 (9)	0.0288 (10)	0.0226 (12)	−0.0037 (8)	0.0058 (8)	0.0051 (8)
O4	0.0198 (10)	0.0308 (11)	0.0220 (12)	−0.0108 (9)	0.0101 (9)	−0.0093 (9)
N1	0.0184 (10)	0.0129 (10)	0.0136 (12)	0.0004 (9)	0.0084 (9)	0.0013 (9)
N2	0.0138 (10)	0.0201 (11)	0.0179 (13)	−0.0030 (9)	0.0070 (10)	−0.0010 (9)
C1	0.0141 (12)	0.0193 (12)	0.0118 (14)	−0.0012 (10)	0.0050 (11)	−0.0017 (10)
C2	0.0147 (12)	0.0179 (12)	0.0106 (13)	−0.0003 (10)	0.0045 (11)	−0.0011 (10)
C3	0.0147 (12)	0.0247 (14)	0.0179 (15)	0.0033 (11)	0.0069 (11)	0.0009 (11)
C4	0.0242 (14)	0.0214 (13)	0.0218 (17)	0.0083 (12)	0.0128 (13)	0.0048 (12)
C5	0.0342 (16)	0.0137 (13)	0.0355 (19)	0.0010 (12)	0.0221 (15)	0.0026 (12)
C6	0.0221 (13)	0.0178 (12)	0.0293 (18)	−0.0016 (11)	0.0173 (13)	0.0020 (11)
C7	0.0200 (13)	0.0223 (13)	0.0164 (15)	−0.0016 (11)	0.0067 (12)	−0.0002 (11)
C8	0.0215 (13)	0.0229 (13)	0.0198 (16)	−0.0015 (12)	0.0110 (12)	0.0002 (12)
C9	0.0161 (12)	0.0232 (13)	0.0221 (16)	−0.0039 (11)	0.0118 (12)	−0.0057 (11)
C10	0.0161 (12)	0.0194 (13)	0.0168 (15)	−0.0009 (11)	0.0074 (11)	−0.0043 (11)
C11	0.0118 (12)	0.0288 (14)	0.0238 (17)	−0.0021 (11)	0.0079 (12)	−0.0063 (12)
C12	0.0153 (13)	0.0290 (14)	0.0182 (16)	0.0006 (11)	0.0017 (12)	0.0000 (12)
C13	0.0180 (13)	0.0210 (13)	0.0167 (15)	−0.0010 (11)	0.0062 (12)	−0.0013 (11)
C14	0.0186 (13)	0.0282 (15)	0.0191 (16)	0.0016 (12)	0.0036 (12)	0.0033 (12)
C15	0.0261 (14)	0.0266 (14)	0.0183 (16)	−0.0019 (12)	0.0087 (13)	0.0041 (12)
C16	0.0175 (13)	0.0250 (14)	0.0217 (17)	−0.0048 (11)	0.0101 (12)	−0.0027 (12)
C17	0.0152 (12)	0.0174 (12)	0.0163 (15)	−0.0022 (10)	0.0044 (11)	−0.0035 (11)
C18	0.0155 (12)	0.0159 (12)	0.0169 (15)	−0.0036 (10)	0.0056 (11)	−0.0038 (11)
C19	0.0134 (12)	0.0160 (12)	0.0150 (14)	−0.0023 (10)	0.0047 (11)	−0.0046 (10)
C20	0.0347 (17)	0.0435 (18)	0.0256 (19)	−0.0095 (14)	0.0175 (15)	−0.0087 (14)

### Geometric parameters (Å, º)

**Table:**

Sn1—O1	2.0949 (15)	C7—C8	1.385 (3)
Sn1—N1	2.2092 (19)	C7—H7	0.9500
Sn1—Cl3	2.3828 (7)	C8—C9	1.374 (4)
Sn1—Cl2	2.3851 (6)	C8—H8	0.9500
Sn1—Cl1	2.3903 (6)	C9—C10	1.403 (4)
Sn1—Cl4	2.4231 (7)	C9—H9	0.9500
O1—C1	1.294 (3)	C10—C19	1.414 (3)
O2—C1	1.220 (3)	C10—C11	1.428 (4)
O3—C17	1.352 (3)	C11—C12	1.347 (4)
O3—H3	0.835 (10)	C11—H11	0.9500
O4—C20	1.431 (3)	C12—C13	1.436 (3)
O4—H4	0.831 (10)	C12—H12	0.9500
N1—C6	1.338 (3)	C13—C14	1.394 (4)
N1—C2	1.342 (3)	C13—C18	1.423 (4)
N2—C7	1.330 (3)	C14—C15	1.377 (3)
N2—C19	1.363 (3)	C14—H14	0.9500
N2—H2	0.870 (10)	C15—C16	1.394 (4)
C1—C2	1.511 (3)	C15—H15	0.9500
C2—C3	1.375 (3)	C16—C17	1.382 (4)
C3—C4	1.381 (4)	C16—H16	0.9500
C3—H3A	0.9500	C17—C18	1.422 (3)
C4—C5	1.378 (4)	C18—C19	1.438 (3)
C4—H4A	0.9500	C20—H20A	0.9800
C5—C6	1.373 (3)	C20—H20B	0.9800
C5—H5	0.9500	C20—H20C	0.9800
C6—H6	0.9500		
			
O1—Sn1—N1	76.37 (7)	C8—C7—H7	119.9
O1—Sn1—Cl3	87.87 (5)	C9—C8—C7	118.5 (2)
N1—Sn1—Cl3	91.69 (6)	C9—C8—H8	120.7
O1—Sn1—Cl2	169.28 (5)	C7—C8—H8	120.7
N1—Sn1—Cl2	92.91 (5)	C8—C9—C10	121.2 (2)
Cl3—Sn1—Cl2	92.68 (2)	C8—C9—H9	119.4
O1—Sn1—Cl1	93.77 (5)	C10—C9—H9	119.4
N1—Sn1—Cl1	168.64 (5)	C9—C10—C19	118.7 (2)
Cl3—Sn1—Cl1	93.58 (2)	C9—C10—C11	122.1 (2)
Cl2—Sn1—Cl1	96.88 (2)	C19—C10—C11	119.2 (2)
O1—Sn1—Cl4	87.18 (5)	C12—C11—C10	120.4 (2)
N1—Sn1—Cl4	83.78 (6)	C12—C11—H11	119.8
Cl3—Sn1—Cl4	173.96 (2)	C10—C11—H11	119.8
Cl2—Sn1—Cl4	91.54 (2)	C11—C12—C13	122.0 (3)
Cl1—Sn1—Cl4	90.20 (2)	C11—C12—H12	119.0
C1—O1—Sn1	118.61 (15)	C13—C12—H12	119.0
C17—O3—H3	112 (2)	C14—C13—C18	119.9 (2)
C20—O4—H4	109.5 (19)	C14—C13—C12	120.6 (2)
C6—N1—C2	119.2 (2)	C18—C13—C12	119.6 (2)
C6—N1—Sn1	127.39 (16)	C15—C14—C13	120.5 (3)
C2—N1—Sn1	113.00 (15)	C15—C14—H14	119.8
C7—N2—C19	124.3 (2)	C13—C14—H14	119.8
C7—N2—H2	120 (2)	C14—C15—C16	120.8 (3)
C19—N2—H2	115 (2)	C14—C15—H15	119.6
O2—C1—O1	124.9 (2)	C16—C15—H15	119.6
O2—C1—C2	119.1 (2)	C17—C16—C15	120.1 (2)
O1—C1—C2	116.1 (2)	C17—C16—H16	119.9
N1—C2—C3	122.0 (2)	C15—C16—H16	119.9
N1—C2—C1	115.6 (2)	O3—C17—C16	122.6 (2)
C3—C2—C1	122.4 (2)	O3—C17—C18	117.0 (2)
C2—C3—C4	118.7 (2)	C16—C17—C18	120.4 (2)
C2—C3—H3A	120.7	C17—C18—C13	118.4 (2)
C4—C3—H3A	120.7	C17—C18—C19	124.0 (2)
C5—C4—C3	119.2 (2)	C13—C18—C19	117.7 (2)
C5—C4—H4A	120.4	N2—C19—C10	117.1 (2)
C3—C4—H4A	120.4	N2—C19—C18	121.7 (2)
C6—C5—C4	119.3 (2)	C10—C19—C18	121.1 (2)
C6—C5—H5	120.4	O4—C20—H20A	109.5
C4—C5—H5	120.4	O4—C20—H20B	109.5
N1—C6—C5	121.6 (2)	H20A—C20—H20B	109.5
N1—C6—H6	119.2	O4—C20—H20C	109.5
C5—C6—H6	119.2	H20A—C20—H20C	109.5
N2—C7—C8	120.1 (3)	H20B—C20—H20C	109.5
N2—C7—H7	119.9		
			
N1—Sn1—O1—C1	−2.17 (18)	N2—C7—C8—C9	−0.6 (4)
Cl3—Sn1—O1—C1	−94.42 (18)	C7—C8—C9—C10	0.1 (4)
Cl2—Sn1—O1—C1	−1.2 (4)	C8—C9—C10—C19	0.7 (4)
Cl1—Sn1—O1—C1	172.13 (18)	C8—C9—C10—C11	−178.4 (2)
Cl4—Sn1—O1—C1	82.12 (18)	C9—C10—C11—C12	179.8 (2)
O1—Sn1—N1—C6	177.6 (2)	C19—C10—C11—C12	0.7 (4)
Cl3—Sn1—N1—C6	−95.0 (2)	C10—C11—C12—C13	0.5 (4)
Cl2—Sn1—N1—C6	−2.2 (2)	C11—C12—C13—C14	179.7 (3)
Cl1—Sn1—N1—C6	147.4 (2)	C11—C12—C13—C18	−1.1 (4)
Cl4—Sn1—N1—C6	89.0 (2)	C18—C13—C14—C15	0.2 (4)
O1—Sn1—N1—C2	4.76 (17)	C12—C13—C14—C15	179.4 (3)
Cl3—Sn1—N1—C2	92.16 (17)	C13—C14—C15—C16	0.1 (4)
Cl2—Sn1—N1—C2	−175.07 (17)	C14—C15—C16—C17	0.1 (4)
Cl1—Sn1—N1—C2	−25.5 (4)	C15—C16—C17—O3	178.8 (2)
Cl4—Sn1—N1—C2	−83.84 (17)	C15—C16—C17—C18	−0.6 (4)
Sn1—O1—C1—O2	−179.9 (2)	O3—C17—C18—C13	−178.5 (2)
Sn1—O1—C1—C2	−0.6 (3)	C16—C17—C18—C13	0.9 (4)
C6—N1—C2—C3	0.0 (4)	O3—C17—C18—C19	1.1 (4)
Sn1—N1—C2—C3	173.5 (2)	C16—C17—C18—C19	−179.4 (2)
C6—N1—C2—C1	180.0 (2)	C14—C13—C18—C17	−0.7 (4)
Sn1—N1—C2—C1	−6.5 (3)	C12—C13—C18—C17	−179.9 (2)
O2—C1—C2—N1	−175.7 (2)	C14—C13—C18—C19	179.6 (2)
O1—C1—C2—N1	4.9 (3)	C12—C13—C18—C19	0.4 (4)
O2—C1—C2—C3	4.3 (4)	C7—N2—C19—C10	0.5 (4)
O1—C1—C2—C3	−175.1 (3)	C7—N2—C19—C18	−179.9 (2)
N1—C2—C3—C4	0.0 (4)	C9—C10—C19—N2	−1.0 (3)
C1—C2—C3—C4	−180.0 (2)	C11—C10—C19—N2	178.1 (2)
C2—C3—C4—C5	0.4 (4)	C9—C10—C19—C18	179.5 (2)
C3—C4—C5—C6	−0.7 (4)	C11—C10—C19—C18	−1.4 (4)
C2—N1—C6—C5	−0.4 (4)	C17—C18—C19—N2	1.7 (4)
Sn1—N1—C6—C5	−172.9 (2)	C13—C18—C19—N2	−178.7 (2)
C4—C5—C6—N1	0.8 (5)	C17—C18—C19—C10	−178.8 (2)
C19—N2—C7—C8	0.3 (4)	C13—C18—C19—C10	0.8 (4)

### Hydrogen-bond geometry (Å, º)

**Table:**

*D*—H···*A*	*D*—H	H···*A*	*D*···*A*	*D*—H···*A*
O3—H3···O4	0.84 (1)	1.73 (1)	2.554 (2)	170 (3)
O4—H4···O2	0.83 (1)	1.87 (1)	2.698 (3)	174 (3)
N2—H2···Cl4^i^	0.87 (1)	2.58 (2)	3.255 (2)	135 (2)

Symmetry code: (i) −*x*+1/2, *y*−1/2, −*z*+3/2.
